# Laparoscopic bile duct surgery: Home truths

**DOI:** 10.4103/0972-9941.30678

**Published:** 2007

**Authors:** Alfred Cuschieri

**Affiliations:** Division of Medical Sciences, Scuola Superiore Sant'Anna di Studi Universitari Pisa, Italy

In this issue of JMAS, the article by Bandyopadhyay, *et al.* on ‘Antegrade common bile duct (CBD) stenting after laparoscopic CBD exploration’[1] makes interesting reading in addition to raising certain unsettled issues concerning this laparoscopic procedure. In the first instance, the authors are to be complemented for the excellent clinical outcome of their patients treated for ductal calculi with no mortality and a morbidity approximating 6%, which is well below that reported in most published series. Another aspect of the series, which is most interesting is the low negative endoscopic retrograde cholangiopancreatography (ERCP) rate, 16/316 patients (5%) contrasting with the reported negative rates varying from 20 to 50%. It is obvious that the authors have very effective protocols for the selection of patients requiring ERCP.

There is good evidence from one large multi-centre RCT[2] and a smaller single-centre RCT[3] together with many large non-randomized series[4–8] that single stage laparoscopic treatment for patients with symptomatic gallstones and ductal calculi is as safe and as effective as two-stage treatment (endoscopic stone extraction followed by laparoscopic cholecystectomy), but it reduces costs and is kinder to the patients. Nonetheless, the two-stage treatment continues to be the most frequent form of management worldwide. The reasons for this anachronistic situation in this era of evidence-based medicine are several: turf battle and dominant hold by gastroenterologists, the hassle factor prevalent amongst many surgeons (cannot be bothered to take on the extra work and training entailed) and several unresolved surgical issues regarding the actual intra-operative management, which are raised by the present publication in JMAS. The important issues regarding the laparoscopic surgical management of ductal calculi include: (i) technique of ductal clearance, (ii) need for drainage of CBD and if so, the best method to achieve this, and (iii) indications for internal bilio-enteric drainage (choledochoduodenostomy/choledochojejunostomy).

## TECHNIQUES OF LAPAROSCOPIC DUCTAL CLEARANCE

The authors of the present series dismiss completely trans-cystic duct exploration and indicate that direct supraduodenal CBD exploration is preferred in their institution as it is more reliable. On the basis of my own experience and published evidence.[6,8] I would have to disagree with this. In the first instance, laparoscopic trans-cystic ductal clearance is infinitely less traumatic than supraduodenal choledochotomy, i.e., it leaves the entire extrahepatic biliary tract in its pristine state and for this reason, recovery from this procedure is almost identical to that of LC alone.[2] It is applicable to about 60% of cases. The size of stone(s) which can be extracted through the cystic duct obviously depends on the size of the cystic duct; although some actually balloon dilate the duct, a practice, which I do not advocate as it may induce splitting of the duct. Obviously there are cases where trans-cystic duct clearance is not applicable - large stones (> 8 mm), occluding stones, excessive stone load in a grossly dilated common bile duct. But in all other instances, the trans-cystic duct clearance (with blind trawling or visually guided through a mini-choledochoscope) should be tried first and direct supraduodenal exploration kept in reserve in the event of failure.

## NEED FOR DRAINAGE OF THE CBD

There is no need for drainage of the CBD after successful trans-cystic ductal clearance as there is no sutured choledochotomy to protect. Most would drain the CBD after successful supraduodenal choledochotomy to ensure decompression, which reduces the risk of post-operative bile leakage. However, not all agree with this and some close the choledochotomy without drainage.[5] These are brave surgeons as flow debimetry studies have shown that there is indeed a temporary period (several days) of impaired emptying through the choledochal sphincter especially after instrumental manipulation inside the CBD.[9] The question is how? It is my strong belief that T-tube drainage should be dropped from surgical laparoscopic bile duct surgical practice as it negates all the advantages of the laparoscopic approach and contributes substantially to the post-operative morbidity.[2] In which case, the options available to the laparoscopic surgeon who believes that decompression is advisable are two: (i) insertion of an endo-biliary stent as reported in this series and used by many other surgeons or (ii) use of a cystic duct drainage cannula (Fr 8) introduced and practised by the author.[10,11] This cannula (Wilson Cook) is inserted into the CBD through the cystic duct to which it is doubly tied with absorbable material. The choledochotomy is then sutured. The cystic duct drainage cannula usually drains approximately 500 mL of bile in first 24 hours after which a post-operative cholangiogram is performed through the cannula. If this is normal, the cannula is capped (Luer-lock fitting cap) and then coiled and covered with an occlusive bandage; the patient being discharged the following day. The cannula is removed as an outpatient 10 days later (longer in diabetics and the elderly). The relative benefits between the two options - temporary stenting versus cystic duct drainage cannula have never been studied but obviously the stent option incurs greater costs because it necessitates a subsequent flexible endoscopy. Other than reduced costs, the other advantage of the cystic duct drainage cannula is that it permits a postoperative contrast study confirming that the ductal stone clearance has indeed been complete.

## INDICATIONS FOR BILIO-ENTERIC DRAINAGE

This was considered necessary in 59 patients in the present series (33%). This is higher than expected from reported series in the West. It may of course indicate more severe and advanced ductal stone disease in India. Either way, it does raise the matter of indications for this added procedure, which are not discussed in the reported laparoscopic surgical literature. In my own practice a bilio-enteric by-pass, usually a transection choledochoduodenostomy, is reserved for patients with ducts exceeding 20 mm and a large stone load. *Am I denying some patients the possible benefit of bilio-enteric drainage or am I being sensible and cautious?*
